# Exergaming in older adults: the effects of game characteristics on brain activity and physical activity

**DOI:** 10.3389/fnagi.2023.1143859

**Published:** 2023-05-05

**Authors:** Helen Müller, Jochen Baumeister, Ellen Marie Bardal, Beatrix Vereijken, Nina Skjæret-Maroni

**Affiliations:** ^1^Exercise Science and Neuroscience Unit, Department of Exercise & Health, Faculty of Science, Paderborn University, Paderborn, Germany; ^2^Department of Neuromedicine and Movement Science, Norwegian University of Science and Technology (NTNU), Trondheim, Norway

**Keywords:** exergaming, EEG, theta, alpha, accelerometer, heart rate, RPE

## Abstract

**Introduction:**

Exergames are increasingly used in rehabilitation settings for older adults to train physical and cognitive abilities. To meet the potential that exergames hold, they need to be adapted to the individual abilities of the player and their training objectives. Therefore, it is important to know whether and how game characteristics affect their playing. The aim of this study is to investigate the effect of two different kinds of exergame (step game and balance game) played at two difficulty levels on brain activity and physical activity.

**Methods:**

Twenty-eight older independently living adults played two different exergames at two difficulty levels each. In addition, the same movements as during gaming (leaning sideways with feet in place and stepping sideways) were performed as reference movements. Brain activity was recorded using a 64-channel EEG system to assess brain activity, while physical activity was recorded using an accelerometer at the lower back and a heart rate sensor. Source-space analysis was applied to analyze the power spectral density in theta (4 Hz–7 Hz) and alpha-2 (10 Hz–12 Hz) frequency bands. Vector magnitude was applied to the acceleration data.

**Results:**

Friedman ANOVA revealed significantly higher theta power for the exergaming conditions compared to the reference movement for both games. Alpha-2 power showed a more diverse pattern which might be attributed to task-specific conditions. Acceleration decreased significantly from the reference movement to the easy condition to the hard condition for both games.

**Discussion:**

The results indicate that exergaming increases frontal theta activity irrespective of type of game or difficulty level, while physical activity decreases with increasing difficulty level. Heart rate was found to be an inappropriate measure in this population older adults. These findings contribute to understanding of how game characteristics affect physical and cognitive activity and consequently need to be taken into account when choosing appropriate games and game settings for exergame interventions.

## 1. Introduction

With aging, the risk of illness and loss of physical function increases, which in turn can lead to cognitive impairment, frailty, and falls (Horak et al., 1989). Approximately one third of adults 65 years of age or older falls once a year (Rubenstein, 2006), making falls the leading cause of injuries in this age group (Sterling et al., 2001). Exercise programs are recommended as preventive measures with good evidence (Gillespie et al., 2012), where greater relative effects were found for exercise programs that challenged balance or used a high dose of exercise (Sherrington et al., 2017). In addition, there is increasing evidence that cognitive problems and particularly poor executive functions are associated with increased risk of falls and should therefore be considered as a training target for fall prevention as well (Mirelman et al., 2012; Kearney et al., 2013).

One way to combine physical exercise with cognitive training is by using exergames. These games require physical activity in an interactive and cognitively demanding digital, augmented, or virtual game-like environment (Stojan and Voelcker-Rehage, 2019). Even though exergames were initially created for entertainment for children and youth, they have become popular as a means to improve physical activity, health and physical function for older adults as well (Brox et al., 2011; Primack et al., 2012; van Diest et al., 2013; Skjæret et al., 2016). Several studies have used exergames to increase general physical activity in older adults (Maillot et al., 2012; Larsen et al., 2013), as well as in specific rehabilitation settings to improve physical function (Baltaci et al., 2013; Hung et al., 2014; Harris et al., 2015). Studies have shown that playing exergames in older adults is effective in increasing balance (van Diest et al., 2013; Donath et al., 2016) and improving stepping (Schoene et al., 2013) and gait parameters (Lee et al., 2014). Furthermore, exergames have been found to be generally as effective as—or more effective than—traditional exercise programs for older adults (Skjæret et al., 2016). Due to the interactive character of the games, not only physical activity of the players is challenged, but also cognitive functions (Anders et al., 2018). Stanmore et al. (2017) showed that exergaming can improve overall cognitive function in older adults, as well as the specific domains of executive functions, attentional processing, and visuospatial skills. The positive effects of exergames on both physical and cognitive functions make exergames a potential intervention for fall prevention (Donath et al., 2016; Choi et al., 2017; Chen et al., 2021; Jacobsen et al., 2021). However, previous studies have used many different exergaming systems and games with different ways to control them. This raises several questions when planning an exergaming intervention: which games should be played, how to set the level of difficulty in the games, and what effects do these choices may have on the players’ brain activity and physical activity during gameplay?

Schoene et al. (2014) classified exergaming interventions into 5 categories depending on the physical activity used: (1) step training, (2) balance board training, (3) balance board plus aerobic training, (4) multi-component programs with low challenge of balance, and (5) aerobic programs. Since exercise programs for fall prevention should include components that challenge balance (Sherrington et al., 2017), the present study focuses on the first two categories.

Balance board training is characterized by exercises with feet in place with only small movements of the center of mass (Schoene et al., 2014). Step training (step-based exergames), where participants are required to take steps in various directions and with various speeds, has been found to reduce falls by approximately 50% (Okubo et al., 2017). Nevertheless, step-based exergames vary in the way they elicit stepping movements, both between and within games, and studies have found that older adults tend to move less with increasing difficulty in the game (Skjæret-Maroni et al., 2016). This is a first indication that the choice of difficulty influences physical activity in terms of amount of exercise.

The positive effects of exergames on cognitive functioning rest on cognitive tests conducted after exergaming as a proxy measure of cognitive processing. Only a limited number of studies have investigated brain activity directly while playing an exergame (Baumeister et al., 2010; Knols et al., 2017; Ko et al., 2020; Ghani et al., 2021; Jacobsen et al., 2021) where electroencephalography (EEG) has been shown to be a valid method to demonstrate the effect of exergames on brain activity (Anders et al., 2018). Previous studies using EEG indicate that exergaming can lead to changes in brain activations, especially in the prefrontal cortex region (Bulea et al., 2017; Knols et al., 2017; Ko et al., 2020; Ghani et al., 2021; Jacobsen et al., 2021). These studies vary widely in population, methodology, exergaming systems, and aim. However, to the best of our knowledge, there is no previous study that has measured EEG during exergaming in the elderly.

In psychological studies, frontal theta and central and parietal alpha frequencies have been described as prominent indicators of cognitive processing in working memory tasks in young adults (Gevins et al., 1997; Sauseng et al., 2005) and older adults (McEvoy et al., 2001). Evidence indicates that frontal theta power is closely related to cognitive demands as theta activity has been found to increase during cognitive testing in young adults and increases further with higher task difficulty (Sauseng et al., 2005; Cavanagh and Frank, 2014). Alpha oscillations are described to be inversely related to neuronal activation in central and parietal brain areas (Pfurtscheller and Lopes da Silva, 1999), which results in decreased alpha activity associated with increased information processing. Furthermore, alpha activity is modulated by the direction of attention and difficulty of the task: external attention (e.g., reading from a screen) causes a decrease in alpha with further decreases during more demanding tasks. In contrast, internal attention (e.g., mental tasks in VR immersion) causes alpha to increase (Magosso et al., 2019).

One of the first studies to describe the neurophysiological processes during exergaming was Anders et al. (2018). This study measured EEG while young healthy participants were playing a balance-exergame that involved solving a jigsaw puzzle. They found increased frontal theta activity during the harder exergame condition compared to both the easier game condition and similar movements made without an exergame context. Furthermore, an increase in central alpha-2 was reported for exergaming conditions compared to the same movement performed in a nongaming context (Anders et al., 2018). To the best of our knowledge, there has been no such study yet on brain activity during exergaming in older adults.

Changing the game and the settings allows to individualize the training to the needs of the player. To do so, it is important to understand how the game characteristics affect the players brain activity and physical activity. Building further on the study of Anders et al. (2018), the aim of the current study is to investigate the effect of the level of difficulty in two different exergames (step game and balance game) on brain activity of older adults in terms of theta power and alpha-2 power, as well as on physical activity in terms of heart rate (HR) and acceleration. Two different exergames that require different movement patterns were played at two different difficulty levels while recording EEG, HR and acceleration. A control condition consisted of making the same movements without an exergame context. We hypothesize that playing an exergame would require increased cortical activity due to external attention as reflected by increased frontal theta activity and decreased alpha-2 activity (Sauseng et al., 2005). By increasing the level of difficulty, we expect to see a further increase in frontal theta and decreased parieto-central alpha-2 activity (Gevins et al., 1997). Regarding physical activity, we hypothesize that older adults move less with increasing difficulty as found previously (Skjæret-Maroni et al., 2016; Skjæret-Maroni and Bardal, 2018), reflected by decreasing acceleration and heart rate.

## 2. Materials and methods

### 2.1. Participants

Twenty-eight healthy older adults participated in the study, recruited by ads in local newspapers. To be included in the study, participants had to be 70 years or older and live independently. Participants were excluded if they had a history of neurodegenerative and/or neurologic diseases, acute physical or mental problems that prevented them from playing an exergame safely for 4 weeks, or had surgery or injury to the back or lower extremities that affected their ability to move pain-free during the study period. The 28 participants (14 female) were 74.47 years old (range: 70–84) with normal to good cognitive and physical abilities and low fear of falling. They did not have experience with exergaming interventions. Most of the participants took one or more of the following medications regularly: Antihypertensives (beta blockers, sodium channel blockers, calcium channel blockers, sartans, ACE inhibitors, acetylsalicylic acid), blood thinners (anticoagulants), diuretics, alpha blockers for prostate enlargement, type II diabetes drugs, analgesics, cholesterol-lowering drugs, and various dietary supplements. Detailed participant information is presented in Table 1.

**Table 1:** Participant characteristics, cognitive ability (MoCA), balance and mobility (CBMS), and fear of falling (FES-I).

	Mean	SE	Min	Max
Age (years)	74.57	0.78	70	84
Height (cm)	172.04	1.86	151	192
Weight (kg)	76.85	2.26	56	102
MoCA	25.04	0.38	21	28
CBMS	68.86	2.03	37	86
FES-I	18.09	0.34	16	22

All participants provided informed, written consent. The study was approved by the Ethics Committee of Paderborn University and was conducted in accordance with the Declaration of Helsinki.

### 2.2. Exergames

**Figure 1:**
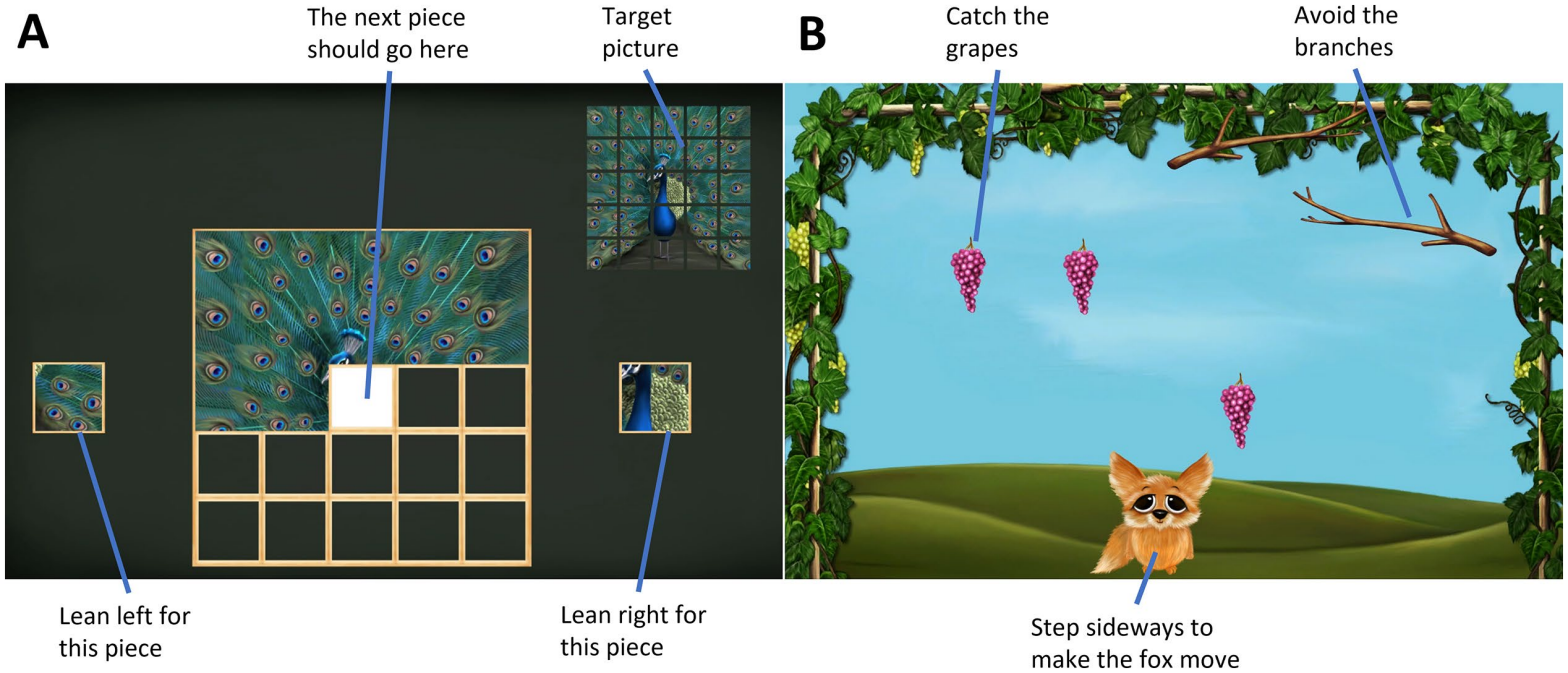
Screen capture of the exergames. **(A)** The puzzle game was controlled by leaning sideways with feet in place; **(B)** the fox game was played by taking steps sideways.

A TV screen-based exergame system (SilverFit BV 3D, Netherlands), which is recommended for balance training in physical rehabilitation of older adults, was used. To control the games, a time-of-flight camera recorded the body movements of the player in three dimensions within a 5 × 5 meter game area, corresponding to a 176 × 144 pixel array (Rademaker et al., 2009). Two different games, puzzle (a leaning game) and fox (a stepping game) (see Figure 1), were played at two levels of difficulty.

The puzzle game consists of a 5 × 5 pieces jigsaw puzzle. Leaning either left or right, the participant selected a puzzle piece. In the easy condition (PE = puzzle easy), only one puzzle piece was shown either on the left-or right-hand side and the participant was required to lean to the side with the puzzle piece. In the harder condition (PH = puzzle hard), the participant had to choose between two puzzle pieces and decided by leaning in the direction corresponding to the correct piece (see Figure 1A). The game is scored based on the time the player uses to solve the puzzle.

In the fox game the participant was required to take sideways steps, thereby controlling the “fox” in the game to catch grapes that were falling from the top of the screen (see Figure 1B; FE = fox easy). In the harder condition (FH = fox hard), additional branches were falling that the players needed to avoid, while still catching the grapes. Arm movements were omitted in favor of better data quality in the EEG, as these would lead to increased muscle activity in the shoulder and neck region, as well as potentially disturb the cables of the EEG system. The speed of the game was set to 3 of 10 for all participants. In the fox, the game score is shown as numbers of grapes caught minus 2 points for each branch that hit the fox. As the number of grapes is lower in FH, the percentage of the total grapes caught was analyzed in addition.

### 2.3. Experimental procedure

All data were collected at the Exercise Neuroscience Lab at Paderborn University. All participants were invited to an information session in the laboratory to familiarize themselves with the equipment and laboratory environment before the actual data collection. On the testing day, cognitive ability (MoCA), mobility and balance (CBMS), and fear of falling (FES-I) were assessed first. The montreal cognitive assessment test (MoCA)^1^ is a one-page 30-point test administered in 10 min assessing short-term memory, visuospatial abilities, executive functions, attention, concentration, working memory, language, and orientation in time and place (Nasreddine et al., 2005). The community balance and mobility scale (CBMS) is used to evaluate higher level balance and mobility on 13 tasks scored from 0 to 5. The items on the CBMS are “unilateral stance,” “tandem walking,” “180-degree tandem pivot,” “lateral foot scooting,” “hopping forward,” “crouch and walk,” “lateral dodging,” “walking & looking,” “running with controlled stop,” “forward to backward walking,” “walk, look & carry,” “descending stairs,” and “step-ups x1.” Higher scores are indicative of better balance and mobility. One item (descending stairs) is scored from 0 to 6, with an extra point given for carrying a basket while descending stairs. The maximum score is 96 points (Weber et al., 2018). Participants also filled out the German version of the 16-item falls efficacy scale-international (FES-I) questionnaire that measures the level of concern for falling on a four-point scale (score16–64) (Yardley et al., 2005).

After finalizing all questionnaires, the participants were equipped with an active 64-channel EEG system, a chest belt for HR measurement connected to a Polar watch and an accelerometer that was placed on the lower back (L3). EEG data, HR and accelerometer data were recorded continuously throughout the entire testing protocol.

Before playing the exergames, participants performed a reference measure for each game by performing the same movements as used in the game (leaning sideways in puzzle, labeled PRef, and stepping sideways in fox, labeled FRef) while looking at a black screen. In the puzzle game, participants were instructed to keep their feet on the spot and tilt their upper body to the left and right as far as possible. For the fox game, they were asked to take steps to the left and right in a range of about 5 m. The movement was demonstrated once by the study leader. Speed or amplitude were not controlled. Subsequently, the participants played both games at two levels of difficulty in a counterbalanced order. In total, the participants played seven puzzle leaning games at each difficulty level and 2 × 4 minutes of the fox stepping game at each difficulty level. After each of the two games, the participants rated the perceived physical and cognitive exertion (RPE) on a visual analog scale (VAS) answering the questions “*How physically exhausting were the games perceived*” and “*How cognitively exhausting were the games perceived?*” with not exhausting at all rated as 1 to totally exhausting rated as 10. In total, the game session lasted approximately 45 min.

### 2.4. Physical activity measurement

Physical activity while playing the exergames was measured using an accelerometer (Sasaki et al., 2018) and a Polar heart rate sensor.

The triaxial accelerometer (AX3, Axivity, United Kingdom) placed on the lower back (L3) was used to assess how much the participants moved while playing the different games. The triaxial raw acceleration from the sensor was summarized to vector magnitude [VM, VM(*g*) = √(*x*^2^ + *y*^2^ + *z*^2^)] using “highpass filtered followed by euclidian norm” (HFEN) method, with a 1 s window length (van Hees et al., 2013). Due to technical problems during data collection and a bad signal-to-noise ratio, seven participants were excluded from these analyzes.

HR data were collected using a chest belt connected to a watch (Polar H10 & Polar M430, Polar Electronics, Kempele, Finland). The mean values were calculated per condition. Due to technical problems during data collection, HR data from 2 participants are missing. Twelve of the remaining subjects used beta-blockers, sodium channel blockers, or calcium channel blockers that are reported to have a putative bias on HR (Aktories et al., 2017). Therefore, the HR data were not further analyzed.

### 2.5. Brain activity measurement

#### 2.5.1. Data acquisition

Brain activity was recorded continuously throughout the session using 64 active electrodes (ActiCap, Brain Products, Germany) and a wireless amplifier (Live Amp 64, Brain Products, Germany). The sampling rate was 500 Hz. EEG electrodes were applied using the international 10–20 system (Klem et al., 1999) with the ground electrode placed mid forehead (Pivik et al., 1993) and referenced online to FCz. Impedance testing ensured a sufficient signal-to-noise ratio in the EEG recording. For standardization purposes, the electrode positions were scanned and saved using the Cap Trak (Brain Products, Germany). Prior to the measurements, the participants were made aware of possible artifacts like blinking, biting teeth or increased muscle activity, with the request to try to keep these artifacts as low as possible. The different conditions in each game were triggered automatically when starting and stopping each game.

#### 2.5.2. EEG analysis

All processing of the EEG data was performed using the EEGLAB toolbox v2020_0 (Delorme and Makeig, 2004) for MATLAB (Version R2019b, Mathworks Inc., Natick, United States).

An EEG processing pipeline has been applied which has been used in previous studies (e.g., Anders et al., 2018; Lehmann et al., 2020) by applying the cleanline plug-in to remove sinusoidal noise (Mullen, 2011). Then, the data was FIR filtered at 3 Hz and 30 Hz. Subsequently, the data were referenced to a common average and downsampled to 256 Hz. Channels that were linked via electrical bridges due to low impedance were detected by the eBridge plugin (Alschuler et al., 2014) and removed. Additional noisy channels were detected and removed with the EEGLAB pop_rejchan function. On average 59.64 (SE 0.51) of the 64 channels were kept per participant for further analysis. For further cleaning of the data, the clean_rawdata EEGLAB plugin (Miyakoshi and Kothe, 2014) was applied. Any channels that contained nonstereotypical artifacts or extreme noise were deleted. The transients of large-amplitude artefacts were interpolated by applying automated subspace reconstruction (ASR). ASR was calibrated on a clean part of the data and a cutoff value of 7 standard deviations (SD) was selected, following previous studies (Nordin et al., 2020; Jacobsen et al., 2021).

The clean signal was decomposed into independent components (IC) using AMICA (Palmer, 2015). For each IC, the corresponding dipoles were calculated using the DIPFIT toolbox (Oostenveld and Oostendorp, 2002) and classified as brain signals or nonbrain signals (muscle activity, eye activity, EKG, line noise, channel noise, other) using the IClabel plug-in (Pion-Tonachini et al., 2019). On average 20.18 (SE 0.94) ICs were labeled as brain activity per participant, 12.25 (SE 1.03) as muscle activity, 3.14 (SE 0.22) as eye activity, 1.82 (0.22) as EKG, none as line noise, 2.96 (SE 0.43) as channel noise and 19.29 (SE 1.46) as other. Only ICs with a probability of being a brain component larger than 90%, located within the head model and with a residual variance (RV%) <15%, were kept for further analysis. For each participant, on average 4.75 (SE 0.45) brain ICs were identified and in total 133 ICs were clustered according to their location and orientation of the dipoles, power spectra, and scalp maps. Dipoles with a standard deviation >3 from the mean dipole of the final cluster were classified as outliers.

The absolute power of functional ICs within a cluster was calculated as the area under the curve (Pivik et al., 1993) in *a priori* defined frequency bands: theta (4 Hz–7 Hz) for the frontal cluster and alpha-2 (10 Hz–12 Hz) for the central and parietal clusters.

The spectral power for each frequency per component in each condition was exported to Excel and the mean spectral power for each frequency band was calculated.

### 2.6. Statistical analysis

Statistical analysis was performed using SPSS Statistics (Version 26, IBM Corp, Armonk, United States). Descriptive analysis of participant characteristics (age, height, weight, MoCA, CBMS and FES-I) was performed.

All parameters were screened for normality of distribution using QQ plots, histograms, and the Shapiro–Wilk test. As the scores of the games and the RPE were not normally distributed, potential differences were tested with the Wilcoxon signed rank test. Furthermore, the absolute power of the measures of cortical activity were not normally distributed, therefore effects of the different games and conditions on cortical activity were assessed using Friedman-ANOVA, with Bonferroni corrections for *post hoc* comparisons.

For acceleration, a two-way repeated measures ANOVA on game (2) × condition (3) was conducted. Mauchley’s test of sphericity was evaluated, and Greenhouse–Geisser correction was applied if needed. Bonferroni corrections were used for *post hoc* comparisons. The significance level was set at *p* < 0.05. One-tailed significant testing was used for paired comparisons and *post hoc* testing. Effect size (*r*) was calculated for significant paired comparisons and *post hoc* tests.

## 3. Results

### 3.1. Game scores

Overall, participants scored differently depending on the game and the level they played. In general, participants needed significantly more time to complete the PH (115.46 s, SE 4.22) compared to the PE game (70.90 s, SE 1.93; *Z* = −4.62, *p* < 0.001, *r* = 0.87). For the fox game, the score was significantly higher in FE (51.648, SE 0.912) than in FH (25.54, SE 0.26; *Z* = −4.62, *p* < 0.001, *r* = 0.87). The same effect was shown for the percentage of grapes caught: (FE: 99.63%, SE 0.22; FH: 97.43%, SE 0.66; *Z* = −2.73, *p* < 0.005, *r* = 0.60).

### 3.2. Rate of perceived exertion

After each game, participants were asked to rate their level of cognitive and physical exhaustion using the VAS. Both games were rated as more cognitively than physically exhausting, but the differences were not significant (Table 2). For both cognitive and physical exhaustion, the puzzle game was rated significantly higher than the fox game (physical: *Z* = −3.17, *p* < 0.001, *r* = 0.60; cognitive: *Z* = −3.70, *p* < 0.001, *r* = 0.70).

**Table 2:** Visual analog scale cognitive and physical exhaustion.

Game	Parameter	Cognitive	Physical	*Z*	*p*
Puzzle	VAS	3.66 (0.39)	3.21 (0.36)	−1.026	0.153
Fox	VAS	2.13 (0.29)	2.07 (0.20)	−2.53	0.400

Mean (standard error) and results of the Wilcoxon signed rank test comparing cognitive exhaustion with physical exhaustion within the games.

### 3.3. Physical activity measured as acceleration

Repeated measures ANOVA revealed a significant interaction effect between game and condition [*F*(1.11,22.25) = 4.864, *p* < 0.05]. To break down this interaction, contrasts compared all conditions to ref. The contrasts revealed significant interactions when comparing ref to the hard condition [*F*(1,20) = 7.13, *p* < 0.05, *r* = 0.51]. The interaction graph shows that these effects reflect that the hard condition lowered the amount of movement significantly more for the fox game than for the puzzle game.

**Figure 2:**
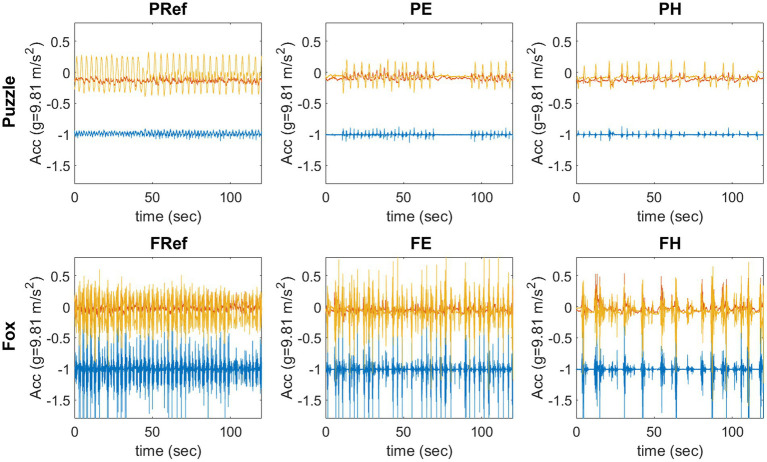
Example of 120 s raw data from one participant in each condition, *x*-axis (vertical) blue, *y*-axis (medio-lateral) yellow, *z*-axis (anterior-posterior) red. Upper row: puzzle game, the participant leaned to the side with feet in place; lower row: fox game, the participant took steps sideways.

**Figure 3:**
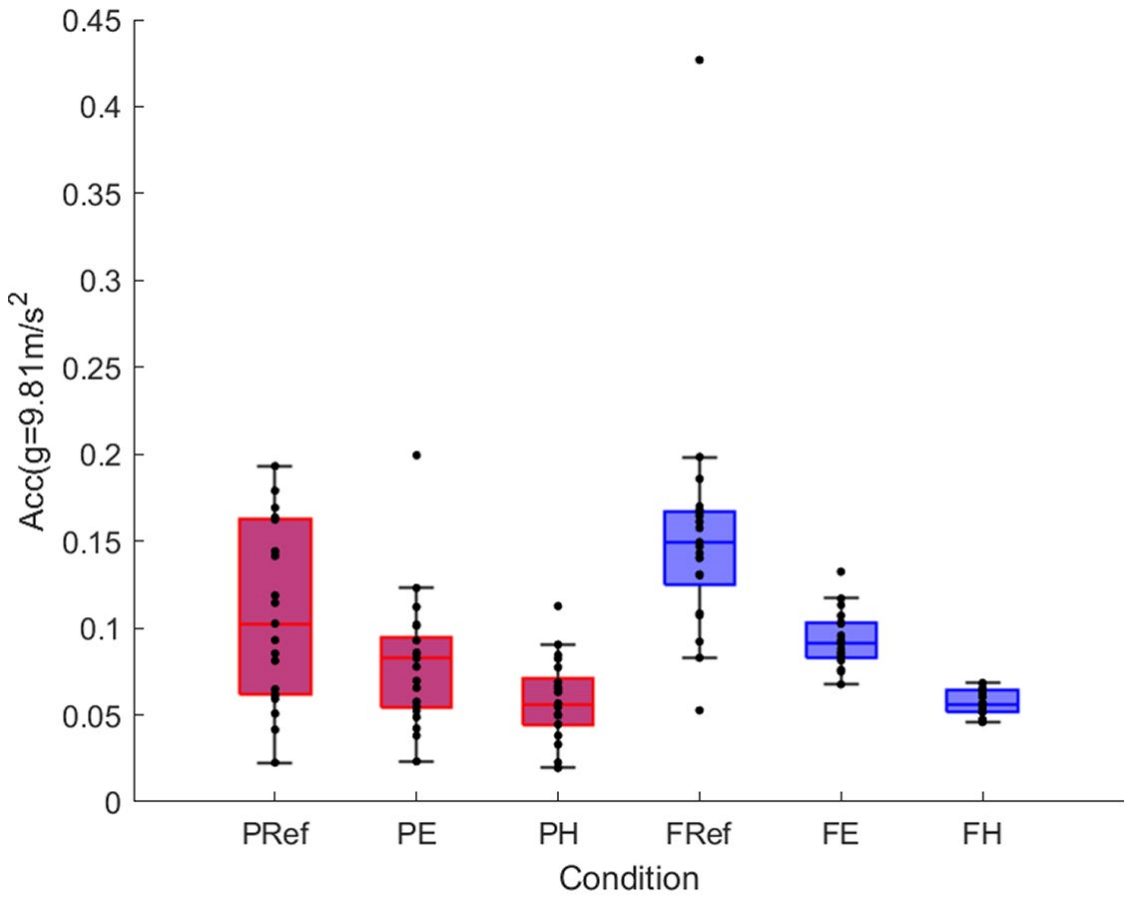
Mean and standard error for physical activity (vector magnitude of acceleration) for puzzle game (red) and fox game (blue) for reference movement, easy and hard conditions.

There was a significant main effect of game [*F*(1,20) = 9.83, *p* < 0.05, *r* = 0.57] and condition [*F*(1.07,21.39) = 47.04, *p* < 0.001], physical activity was significantly higher for the fox (mean difference 0.019 g, 95% CI 0.006–0.032) compared to the puzzle (Figure 2). All conditions were significantly different from each other, with highest physical activity for the reference movements and the lowest for the more challenging conditions (ref-easy *p* < 0.001, *r* = 0.76; ref-hard *p* < 0.001, *r* = 0.86; easy-hard *p* < 0.001, *r* = 0.94; Figure 3).

### 3.4. Brain activity measured with EEG

**Figure 4:**
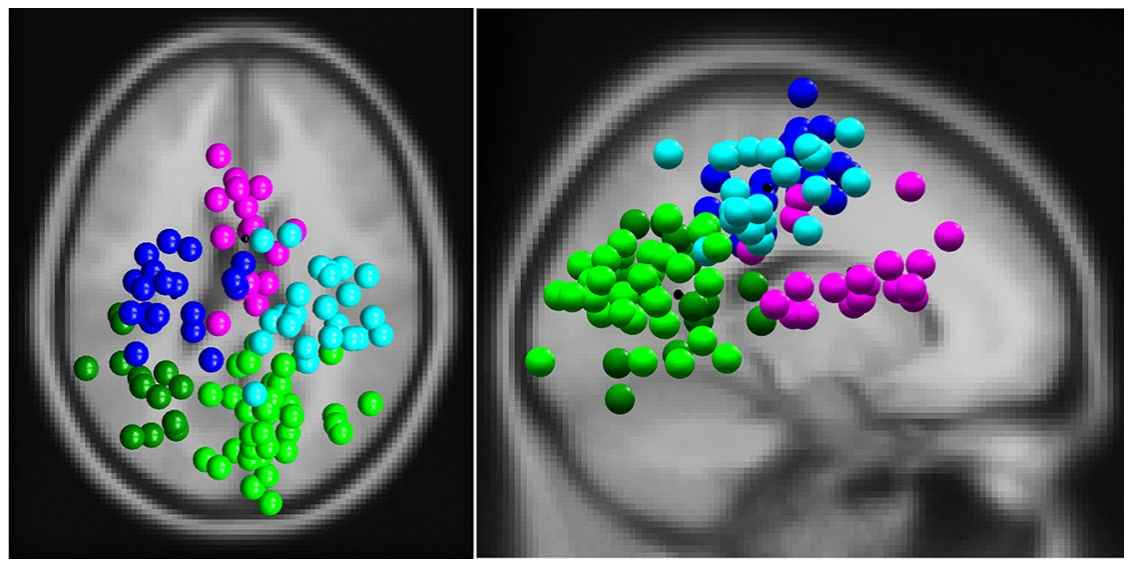
The clusters of independent components (ICs) in a top view (left) and sagittal view (right). ICs were divided into a frontal cluster (magenta, 16 subjects, 19 ICs, RV: 3.21%), two central clusters (left: blue, 16 subjects, 19 ICs, RV: 4.25%; right: cyan, 19 subjects, 24 ICs, RV: 4.27%) and two parietal clusters (left: dark green, 12 subjects, 14 ICs, RV: 3.26%; right: light green, 25 subjects, 32 ICs, RV: 3.72%).

EEG analysis revealed five different clusters: one frontal cluster, two central clusters, and two parietal clusters. Their locations are shown in Figure 4.

For each cluster, the conditions reference movement, easy, and hard, were compared separately for each game. Friedman ANOVA revealed significant effects for all clusters and their assigned frequencies except for central right alpha-2 power (see Table 3).

**Table 3:** Mean (standard error) of the cortical activity and the results of the Friedman ANOVA comparing the three conditions ref, easy, and hard.

Cluster (frequency)	Game	Ref	Easy	Hard	Df	*χ* ^2^	*p*
Frontal (theta)	Puzzle	28.75 (0.28)	29.19 (0.29)	29.22 (0.29)	2	24 20.316	**<0.001**
	Fox	28.99 (0.26)	29.35 (0.31)	29.27 (0.29)	2	13.368	**0.001**
Central left (alpha-2)	Puzzle	23.86 (0.30)	23.91 (0.33)	23.79 (0.31)	2	6.421	**0.040**
	Fox	24.13 (0.39)	23.82 (0.29)	23.92 (0.32)	2	0.737	0.692
Central right (alpha-2)	Puzzle	23.75 (0.46)	23.57 (0.50)	23.62 (0.46)	2	0.250	0.882
	Fox	23.21 (0.48)	23.28 (0.48)	23.25 (0.53)	2	1.583	0.453
Parietal left (alpha-2)	Puzzle	27.45 (0.84)	26.77 (0.82)	26.37 (0.83)	2	9.143	**0.010**
	Fox	27.64 (0.94)	27.08 (0.84)	27.02 (0.91)	2	11.286	**0.004**
Parietal right (alpha-2)	Puzzle	27.85 (0.15)	28.26 (0.19)	28.14 (0.19)	2	30.722	<**0.001**
	Fox	27.92 (0.16)	27.83 (0.18)	28.18 (0.19)	2	32.667	<**0.001**

Bold values are indicating significant values.

#### 3.4.1. Theta activity

Frontal theta power demonstrated a significant main effect of condition in both games. As shown in Table 3, the reference movements showed an overall lower frontal theta power compared to the two gaming levels in both the puzzle (PRef < PE *z* = −1.21, *p* = 0.001, *r* = 0.28; PRef < PH *z* = −1.32, *p* < 0.001, *r* = 0.30) and the fox game (FRef < FE *z* = −1.00, *p* < 0.005, *r* = 0.23; FRef < FH *z* = −1.05, *p* < 0.005, *r* = 0.24).

#### 3.4.2. Alpha-2 activity

For the puzzle game, alpha-2 power showed a significant main effect of condition in both the central and parietal areas (Table 3). *Post hoc* tests did not show any significant differences for the central region.

For the left parietal cluster, a significantly lower power of alpha-2 in PH compared to PRef (*z* = −1.14, *p* < 0.005, *r* = 0.31) was found. In the right parietal cluster, the alpha-2 frequency was significantly lower at PRef compared to PE (*z* = −1.31, *p* < 0.001, *r* = 0.22) and PH (*z* = −0.61, *p* < 0.05, *r* = 0.10). Furthermore, alpha-2 power in PE was higher compared to PH (*z* = 0.69, *p* < 0.010, *r* = 0.12).

For the fox game, there was a significant main effect of condition on the parietal brain areas (Table 3). For the left parietal cluster, a significant *post hoc* difference was found with a higher alpha-2 power for FRef compared to FE (*z* = −0.93, *p* < 0.05, *r* = 0.25) and FH (*z* = 1.21, *p* < 0.005, *r* = 0.32). In the right parietal cluster, alpha-2 power was lower for PRef in comparison to FH (*z* = −0.83, *p* < 0.005, *r* = 0.14). Furthermore, a higher power was found for FH compared to FE (*z* = −1.33, *p* < 0.001, *r* = 0.22).

## 4. Discussion

The present study investigated the effect of game characteristics on brain activity and physical activity of older adults while playing two exergames. The game score was significantly higher in the easy condition than in the hard condition for both games. This indicates that the additional cognitive element in the hard level indeed made it harder than the easy condition. Playing the puzzle and fox games was associated with an overall increase in theta power under easy (PE, FE) and hard (PH, FH) conditions as compared to making the reference movements performed without an exergaming context (PRef), while the alpha-2 power showed a more diverse pattern. Physical activity decreased with increased difficulty as indicated by lower acceleration during both games.

### 4.1. Physical activity during exergaming

One purpose of exergaming is to encourage the player to perform bodily movements and hence be physically active. In this study, the amount of movement that was used when playing the game was measured by an accelerometer. We found that the participants moved considerably more when playing the fox than when playing the puzzle game. Interestingly, perceived rate of exertion showed a discrepancy since participants rated the puzzle game as more physically exhausting than the fox game. Previous studies have shown that RPE increased significantly from resting (Ogawa et al., 2019). Furthermore, Mullins et al. (2012) showed that the results of RPE were mirroring the intensity of the exergames, in that the participants rated the aerobic and strength games significantly higher than yoga and balance games (Mullins et al., 2012). However, our study is not directly comparable as the fox game was played at a relatively slow speed (3 of 10). The higher RPE ratings for the puzzle game may be due to the unusual movement of leaning sideways without stepping sideways, as they do in the fox game, which is a more common movement in everyday life. Observation during gameplay also revealed that the participants found it more difficult to learn the movements of the puzzle game than that of the fox game. Keeping the feet in place and controlling the game simply by leaning the body to the side was not always intuitive for the participants. This could be an explanation for the increased RPE values. Repeated play over time would give participants the chance to become more familiar with the movements, and hence, could change this effect.

However, comparing the three conditions within one game showed that the amount of movement decreased significantly from leaning and stepping movements without a game context, to the easier exergaming conditions and further to the harder version of the games. The raw data (Figure 2) of the physical activity monitoring illustrate this with a comparable amplitude of the movement between all conditions, but a decreasing movement frequency. In contrast to the self-paced movements performed as reference movements, when playing the games, the participants had to react to the game and wait for the next stimulus. This illustrates that physical activity is influenced by the design of the game. One example of this can be found in the harder condition of the fox game where participants should avoid falling branches while still needing to catch the grapes. This often led to breaks in the movements as the participants were waiting for the branches to reach the bottom of the screen, thereby moving less than in the easier condition with grapes only. A similar finding was previously reported for other stepping games as well where adding several elements to the game reduced the overall movements made by the participants (Skjæret-Maroni et al., 2016; Skjæret-Maroni and Bardal, 2018). Decreasing physical activity when adding cognitive elements to the game, and thus increasing the difficulty level, seems to be a robust effect for stepping games. Similar findings for the puzzle game in the current study indicate that this effect can be found for leaning games as well.

Originally, we wanted to include HR as a measure of intensity of physical activity. However, HR turned out not to be a good measure for this population as it does not tell a reliable story in this group. Despite only including older adults without acute diseases, more than half of the participants used one or several medications that are known to affect HR. Antihypertensives are particularly widely used in this population. Earlier studies confirm that HR is not an adequate index of exercise intensity if participants take beta-blockers (Izquierdo et al., 2021; Jacobsen et al., 2021) since participants have a significantly lower resting HR and maximal HR with beta-blockers than without beta-blockers (Arena et al., 2010; Han et al., 2022). Hence, studies should interpret HR findings with caution, and need to report on types of medications used in order to interpret results of exergame activity.

### 4.2. Brain activity during exergaming

The results of the current study showed that frontal theta increased from reference movement to gaming situation, regardless of the type of game and the level of difficulty. Increased frontal theta activity is associated with cognitive demands in the prefrontal cortex, which is often described as reflecting attentional control processes (Gevins et al., 1997; McEvoy et al., 2001; Sauseng et al., 2005).

The increased activity of the frontal theta in the current study indicates that attention and cognitive demand are required while playing exergames. Previous studies have also shown that exergaming required greater attention than performing movements without an exergaming context (Ko et al., 2020). The close relation between frontal theta power and attentional processes has been shown in numerous studies, indicating an association of theta frequency with attention in cognitive (Sauseng et al., 2010; Cavanagh and Frank, 2014) and sensorimotor tasks (Baumeister et al., 2008; Büchel et al., 2021; Jacobsen et al., 2021). Results from previous studies linked frontal theta to the anterior cingulate cortex (ACC) (Gevins et al., 1997; Petersen and Posner, 2012; Ishii et al., 2014). Neuroimaging and brain lesion studies have shown that this anatomical region is an important component of the human attentional system (Karni et al., 1998; Luks et al., 2002). Similar increased frontal activity has been demonstrated also in young adults when playing an exergame (Anders et al., 2018) and has been discussed as reflecting attentional control processes.

Playing an exergame is more than performing a specific movement, since the participant needs to interact with the game by performing the movement correctly and well timed with the game. For instance, to control the fox game, the participant needs to coordinate the sideways steps while paying attention to the game to detect the falling grapes and planning the next sideways steps to reach the grapes before they hit the ground. This inherent dual task of playing an exergame also seems to be cognitively demanding for older adults, as shown by increased frontal theta. Interestingly, this effect occurred regardless of type of movement needed to play the game. An increase in theta has also previously been shown in older adults when performing dual tasks standing on a sway platform (Ozdemir et al., 2016) or in semi tandem stance (Bohle et al., 2019) compared to single task conditions.

In our study, the level of difficulty did not demonstrate a significant effect on frontal theta in either of the games. This is not in line with previous studies that showed increased frontal theta activity with increasing task difficulty (Sauseng et al., 2005; Cavanagh and Frank, 2014). For example, Anders et al. (2018) demonstrated increased frontal activity for the harder puzzle game in young adults compared to the easy condition, although there was no difference between the reference movement and the easy condition.

There is growing evidence that frontal theta increases with difficulty in young adults but not in older adults when testing working memory. Similar findings exist for coordination tasks where frontal theta increases from a simple to a complex coordination task in young adults, but not in middle aged and older adults (Depestele et al., 2023). According to the compensation-related utilization of neural circuits hypothesis—CRUNCH (Reuter-Lorenz and Cappell, 2008), older brains must work harder to reach similar results as young brains. Processing inefficiencies appear in older adults that are likely due to compensation of ongoing decline, noise and irrelevant information that is less inhibited. When solving a cognitive task, more neural resources are recruited in older adults than in young adults to achieve equivalent results. This compensation is effective at lower levels of difficulty, but with increasing demands, the limit of resource capacity might be reached leading to insufficient processing and declined results in the older adults (Reuter-Lorenz and Cappell, 2008). Following this reasoning, our results indicate that the neural capacity might be reached already in the easy games, without much opportunity for frontal theta to increase further in the harder condition.

Despite the lack of a significant increase of frontal theta power with increased difficulty level, both versions of the games in the current study showed increased frontal theta activity when compared to the reference movements. These current findings support that exergames by default require increased cognitive processing also in older adults, as previously shown for younger adults (Anders et al., 2018). In that respect, frontal theta power might serve as a potential biomarker of cognitive processing in exergaming, independent of the type of game or the level of difficulty.

Although the increase in theta power under the exergaming conditions was a robust finding for both exergames, the alpha-2 results show a diverse and less consistent pattern. For the puzzle game, we found an increase in the right parietal area from PRef to PE and PH with a concurrent decrease in the left parietal hemisphere for the PRef to PH. For the fox game, the results were not consistent as we found an increase in alpha-2 power in the right hemisphere only for FH compared to PRef and a decrease for both FE and FH compared to FRef. Activity in the alpha band is described to be inversely related to cognitive demands, and is often found to decrease in cognitive tasks (Sauseng et al., 2005). This effect has also been shown previously for dual task conditions in older adults (Bohle et al., 2019). These findings have led to the hypothesis that decreases in parietal alpha-2 power might be related to increased information processing (Klimesch, 1996) whereas increased alpha-2 activity may indicate cortical inhibition (Klimesch et al., 2007). Anders et al. (2018) found increasing central alpha-2 activity when comparing the reference movement with both difficulty levels of the puzzle game in young adults. They interpreted this as a shift from conscious movements (reference movement) to automated movements during the exergaming with a shift of alpha-2 activity to brain areas that are needed to handle the increased cognitive demand that was added through the exergaming. In contrast, our findings did not show significant changes in the central areas.

The inconsistent dynamics of the alpha-2 frequency might be due to the selection of the upper band as fixed frequency (10 Hz–12 Hz), which was based on previous literature (e.g., Anders et al., 2018). The age of the participants might influence the results since peak alpha frequency decreases with age (Klimesch, 1999). In addition, the alpha peak frequency is further affected by individual states such as diseases, cognitive or physical activity, or emotions, which suggests that the individual alpha peak frequency increases in an activated state (e.g., sensory and motor processing) and decreases when in deactivated state (e.g., resting or meditation) (Mierau et al., 2017). Individual alpha peak analysis could be an alternative way to analyze EEG data in future studies.

Overall, these inconsistent results in alpha-2 power in the two exergames may indicate that this frequency reflects a task-specific pattern for each game. Thus, although it might be possible to describe the changes within a game, these changes may not necessarily transfer to other games or exergaming in general.

### 4.3. Implications for applied settings

In order to implement exergames effectively in applied settings, it is crucial to understand how different movement patterns required to play the games and difficulty levels influence the participants’ brain activity and physical activity. The results of the current study indicate that playing an exergame affects both physical activity expressed as movement acceleration and cortical processing as demonstrated by frontal theta brain activity. Physical activity decreased significantly with increased difficulty, but frontal theta did not increase with higher levels of difficulty. Hence, choosing the right game and appropriate game settings is crucial for the successful application of exergames. If the goal of using an exergame is to become more physically active in general, a stepping game at the easy level might be more suitable than a cognitively more complex version with additional game elements. Furthermore, cognitive processing seems to be an inherent part of exergaming, irrespective of the specific type of movement or the level of difficulty.

For effective implementation of exergaming interventions in rehabilitation, it is important to understand how the games affect brain activity and physical activity. Physical activity revealed robust findings for both games, with a decrease in the acceleration and thus the amount of movement resulting from an increase in the level of difficulty, which is in line with the results of previous studies (Skjæret-Maroni et al., 2016; Skjæret-Maroni and Bardal, 2018). HR is not recommended to use as a measure of activity in this age group if participants use chronotropic medication. Regarding cognitive demands, frontal theta activity showed robust findings for both games, with increased activity for the gaming conditions compared to the reference movements. This indicates that frontal theta might be a potential biomarker to describe cognitive processing in future studies.

### 4.4. Limitations

Some methodological limitations should be considered when interpreting the present findings. A methodological issue inherently related to EEG assessments is the limited spatial resolution (Mehta and Parasuraman, 2013). Although ICA was applied to reduce volume conduction effects, IC dipoles only display an approximation of the real cortical source of the signal and exact spatial assignment of EEG signals should be considered with caution (Jungnickel and Gramann, 2016). For this study, the data was cleaned automatically using artefact subspace reconstruction. However, the ASR does not only detect non-stereotypical artifacts like movement artifacts, but also eye blinks as repetitive and stereotypical artifacts that appear in the frontal channels in particular. Interpolation of these channels might affect frontal brain activity.

During game play, there are phases that require cognitive decisions and phases with less cognitive involvement. For example, in the puzzle game, the participant is solving a puzzle of 25 pieces for 1–2 min. This means 25 decisions, but with several short phases of waiting in between those decisions for feedback from the game and presentation of the next piece of the puzzle. This study analyzed the EEG data with power analysis, which means that one mean value is taken over the entire puzzle game condition. Therefore, the different stages of cognitive involvement could blur the effects. To avoid this, an event-related analysis that only analyzes the short period of time around deciding for each puzzle piece could be an option. However, for an event-related approach, a high number of events is required, which often leads to fatigue and potential adverse events in the participants. As the current study involved older adults, an event-related approach would not have been optimal.

Due to the chronotropic medication of many participants in this population, we did not analyze the HR data further. In addition, the potential influence of medications on brain activity cannot be completely ruled out. As participants with neurological diseases were excluded from this study, no specific neurological medications were taken. However, potential side effects of other drugs cannot be excluded.

To standardize the study, all participants played all games with the same game settings. For the fox game, the game speed was quite low, which resulted in a ceiling effect in the game score: many participants caught all or nearly 100% of the grapes in the easy condition and only slightly fewer in the harder condition. This indicates that participants likely did not need to perform at their maximum potential. Nevertheless, playing the game could show effects both in the amount of movement and in brain activity. To achieve specific training effects from exergaming, it is important that the level of difficulty is customized to the ability of the participants. This should be taken into account in future studies in order to demonstrate best effects for the participants.

One reason why the study could find effects of exergaming on frontal theta activity despite this ceiling effect in the game score could be the novelty effect. Most of the participants had not played an exergame before, so this novelty effect might appear in the EEG recordings. Therefore, further studies should investigate the longitudinal effect of playing exergaming to investigate whether the effects of exergaming on cortical activity reflected by increased frontal theta are maintained over time. In case it is not, individualizing the level of difficulty for each participant over prolonged playing time might be necessary to maintain the cognitive effect.

## Conclusion

This study showed that exergaming inherently increases cognitive activity. The findings demonstrate that frontal theta activity shows a consistent increase in older adults playing exergames compared to doing self-paced movements, regardless of the type of game. Alpha-2 on the other hand, showed inconsistent, potentially task-specific results, indicating that the transfer of those results to other games might be less straightforward.

Physical activity showed an inverse effect on frontal theta activity, namely a decrease while exergaming compared to the same movements without an exergaming context and a further decrease when additional cognitive elements were added to the game. HR data should be interpreted with care in the older population due to likely biased results that can be caused by medications.

However, the specific type of movements used in the exergames did not influence brain activity or physical activity, since the effects were the same for both the fox game and the puzzle game.

This study provides a first insight into the effects that different levels of difficulty have on brain activity and physical activity in older adults playing two different exergames. These findings should be taken into account when choosing the most appropriate game and game settings for an intervention.

## Data availability statement

The raw data supporting the conclusions of this article will be made available by the authors, without undue reservation.

## Ethics statement

The studies involving human participants were reviewed and approved by Ethics Committee of Paderborn University. The patients/participants provided their written informed consent to participate in this study.

## Author contributions

HM, JB, BV, and NS-M contributed to the conception and design of the study. HM collected the data. HM and EB processed the data. HM and NS-M wrote sections of the manuscript. All authors contributed to the article and approved the submitted version.

## Funding

This research was partly funded by grant 57525213 from the German Academic Exchange Service awarded to JB and BV.

## Conflict of interest

The authors declare that the research was conducted in the absence of any commercial or financial relationships that could be construed as a potential conflict of interest.

## Publisher’s note

All claims expressed in this article are solely those of the authors and do not necessarily represent those of their affiliated organizations, or those of the publisher, the editors and the reviewers. Any product that may be evaluated in this article, or claim that may be made by its manufacturer, is not guaranteed or endorsed by the publisher.

## Footnotes

^1^ www.mocatest.org
